# Bacterial community and diversity in the rumen of 11 Mongolian cattle as revealed by 16S rRNA amplicon sequencing

**DOI:** 10.1038/s41598-024-51828-8

**Published:** 2024-01-18

**Authors:** Yijiu Jia, Yali Shi, Huiyan Qiao

**Affiliations:** College of Chemical Engineering, Inner Mongolia University of Technology, No. 49 Aimin Street, Xincheng District, Hohhot, 010051 China

**Keywords:** Applied microbiology, Bioinformatics

## Abstract

Through microorganism in the rumen of ruminant, plant fiber can be converted to edible food such as meat and milk. Ruminants had a rich and complex microbial community within the rumen, and the bacteria comprised the dominant proportion of the ruminal microbes. High-throughput sequencing offered a viable solution for the study of rumen microbes. In this study, rumen fluid samples were taken from 11 cattle from Inner Mongolian, the DNA of 11 rumen fluid samples were extracted and bacterial amplicons of the V4 regions of 16S rRNA were subjected to Illumina sequencing. More than 90,000 raw reads and 60,000 effect Tags per sample were obtained. 28,122 operational taxonomic units (OTUs) were observed from 11 samples, in average 2557 ± 361 OTUs for each sample. *Bacteroidetes* (44.41 ± 7.31%)*, Firmicutes* (29.07 ± 3.78%), and *Proteobacteria* (7.18 ± 5.63%) were the dominant phyla among the bacteria of rumen, accounting for 82%. At the genus level, the highest relative abundance was *Prevotella.* Their functions were predicted using the Kyoto Encyclopedia of Genes and Genomes (KEGG). The results showed that they included metabolism, genetic information processing, environmental information processing and cellular processes. It explored the bacterial community diversity and composition of the rumen of Mongolian cattle. On the whole, our research showed that there was a high diversity as well as rich bacterial flora function of rumen bacteria in Mongolian cattle. Meanwhile, these findings provided information for further studies on the relationship between the community, diversity, functions of rumen bacteria and the nutritional physiological functions of the host.

## Introduction

Ruminants were the source of dairy milk and meat. As an herbivore, the functionality of their digestive system could convert plant fiber into volatile fatty acids and other products. This capacity was of enormous significance to man, and its realization depended on the microorganism of ruminants. About 75% of the fiber in the feed were digested in the rumen^1^. Rumen was a huge and complex microbial ecosystem, which depended on the joint action of bacteria, fungi, protozoa and other microorganisms. Among them, bacteria were the main component of rumen microorganisms, representing about 95% of the total microbes^2^. The rumen microorganisms served for the host as nutrition, immune regulation and physiological processes^3^. The composition and function of rumen microorganisms were not static, they would change with host, environment, diet and other factors.

As an anaerobic environment, only about 20% of rumen microorganisms were cultured conventionally in the laboratory and observed under microscope. It was possible to study microbial diversity, abundance and function for the uncultured microorganisms through metagenomics sequencing without culture. The 16S rRNA gene was an important symbol for a unique microbial. As it included nine hypervariable regions in the gene sequence, and the sequences of the hypervariable regions in a 16S rRNA gene correspond to a unique bacterium^4^. Consequently, the 16S rRNA gene amplicon sequencing was a vital tool to study the communities and diversity of bacteria in rumen^5,6^. The 16S rRNA gene high-throughput sequencing was used for the microbial of the gastrointestinal in 2005 for the first time. It provided a new method for the identification of the uncultured microbial^7^. In order to study the changes of bile acid and intestinal microflora in the perinatal period of Sha ling sows. Jie Wang^8^ carried out 16S rRNA sequencing and bile acid targeted metabolome detection on the feces of 42 sows. The results showed that there was more much bile acid when the intestinal microflora was increasing in the perinatal period of Sha ling sows. Dong SX^9^ used 16S rRNA sequencing on the intestinal microorganisms of wild striped goose in Tibet Autonomous Region. A total of 513,505 original data reads were obtained. The results showed that the phylum Chlamydomonas (67.34%) was the dominant phylum, followed by Proteobacteria (29.03%) and Cyanophyta (1.97%). The findings were also very useful and valuable for the study of genetics and high altitude in Tibet Autonomous Region.

Mongolian cattle are mainly fed in the north of China, Mongolia and the east of Russia^10^, where the altitude here is high, and the weather was cold and dry. Mongolian cattle are hardy, disease resistant, adaptable to harsh environmental conditions and have relatively low food requirements, being able to digest roughage with a high fibre content. It is a treasure of microbial community research because of its unique living environment setting and eating habits^11^.

In order to study the composition and structure of microorganisms in the rumen, we analyzed the sequencing results of the bacterial amplicons of the V4 regions of 16S rRNA of 11 Mongolian cattle rumens. The findings could be helpful for the study of rumen microbial diversity of Mongolian cattle, and they could provide basis for future research on rumen bacteria of Mongolian cattle. Based on the experimental results, the functional bacteria producing cellulase or ethanol was screened from the rumen of Mongolian cattle. In addition, understanding the characteristics of Mongolian cattle rumen microorganisms could provide guidance for their feeding.

## Methods

### Sample collection

A total of 11 Mongolian cattle with two years old were used in this study. And these cattle were originated from Zhenglan Banner, XilingGol League, Inner Mongolia, in China (115°00′-116°42′E, 41°56′-43°11′N). All the animals were raised under the same conditions. The experimental cattle were slaughtered in the same abattoir, where 11 rumen fluid samples were collected immediately. It was necessary to wear sterile gloves when collecting rumen fluid samples. Rumen fluid (approximately 50 mL) was strained through four layers of cheesecloth and centrifuged at 13,000 × g for 20 min at 4 °C to collect the supernatant. Samples were rapidly frozen in liquid nitrogen and stored at − 80°C. They were labeled as N1 to N11.

### Methods

#### Genome DNA extraction and PCR amplification

Total genome DNA of rumen fluid from 11 samples was extracted by Genome DNA Extraction Kit (Qiagen, Germany). The concentration and purity of the genome DNA were monitored using 1% agarose gels. For the procedure of Polymerase Chain Reaction (PCR), DNA was diluted to 1 ng  µL^−1^ using sterile water according to the concentration.

Specific primers 515F-806R were used to amplify the 16S rRNA gene of the 16S V4 region. All PCR reactions were carried out with 15 µL of Phusion® High-Fidelity PCR Master Mix (New England Biolabs). In each reaction forward and reverse primers were 2 µM, and template DNA was 10 ng. Thermal cycling was followed below: an initial denaturation at 98 °C for 1 min; 30 cycles of denaturation at 98 °C for 10 s, annealing at 50 °C for 30 s, and elongation at 72 °C for 30 s; 72 °C for 5 min. The concentration and purity of the PCR products were monitored on 2% agarose gel. The mixture of PCR products was purified with Qiagen Gel Extraction Kit (Qiagen, Germany).

#### 16S rRNA gene amplicon library preparation

Genome DNA libraries of 11 rumen fluids were generated using TruSeq® DNA PCR-Free Sample Preparation Kit (Illumina, USA) and index codes were added. At last, 11 libraries were sequenced through an Illumina NovaSeq platform. 250 bp paired-end reads were generated after sequencing and were named raw data.

#### Processing of sequencing data

The raw data were spliced and controlled on quality to obtain clean tags. Chimerism were filtered to obtain effective tags. The effective tags were clustered with 97% identity and named OTUs (operational taxonomic units). Representative sequences for each OTU were screened and annotated taxonomic information according to Silva Database (http://www.arb-silva.de/)^12^. MUSCLE (Multiple Protein Sequence Alignment) software (Version 3.8.31, http://www.drive5.com/muscle/) was used to analyze the relationship of different OTUs and the dominant species among the 11 samples^13^.

#### Alpha diversity

For studying the species diversity of the 11 samples, alpha diversity was used on 6 indices, including Observed-species, Chao1, Shannon, Simpson, ACE, and Good-coverage. The 6 indices of the 11 samples were calculated with QIIME (Quantitative Insights into Microbial Ecology) (Version 1.7.0) and displayed with R software (Version 2.15.3). The Chao1 estimator (http://www.mothur.org/wiki/Chao) and the ACE estimator (http://www.mothur.org/wiki/Ace) were selected to identify community richness of the 11 samples. The Shannon index (http://www.mothur.org/wiki/Shannon) and the Simpson index (http://www.mothur.org/wiki/Simpson) were used to identify the community diversity of the 11 samples. The Good’s coverage (http://www.mothur.org/wiki/Coverage) was used to characterized Sequencing depth of the 11 samples.

#### Beta diversity

In order to analyze the species complexity among the 11 samples, beta diversity analysis was used. Beta diversity included weighted and unweighted unifrac. Weighted and unweighted unifrac were calculated using QIIME software (Version 1.9.1).

Principal Coordinate Analysis (PCoA) was a visualized form of the sequencing data. In the principal coordinate analysis, a set of orthogonal axe was obtained from a distance matrix of weighted or unweighted unifrac among the 11 samples. The maximum variation factor was demonstrated by first principal coordinate, and the second maximum one by the second principal coordinates, and so on. PCoA analysis was displayed by WGCNA (Weighted correlation network analysis) package, stat packages and ggplot2 package in R software (Version 2.15.3).

Unweighted Pair-group Method with Arithmetic Means (UPGMA) clustering was performed as a type of hierarchical clustering method to interpret the distance matrix using average linkage and was conducted by QIIME software (Version 1.9.1).

#### Function prediction

Kyoto Encyclopedia of Genes and Genomes (KEGG) pathways was a group of pathways showing the relation networks for: 1. Metabolism 2. Gene information transduction 3. Environmental information processing 4. Cell signal transduction 5. Organism Systems 6. Human diseases 7. Drug development.

KEGG pathways were built using the Tax4Fun program. The Tax4Fun function prediction was analyzed by the nearest neighbor method based on the minimum 16S rRNA sequence similarity. The procedure of the Tax4Fun function prediction was followed below: firstly, extracting the whole genome 16S rRNA gene sequences of prokaryotes in KEGG database (www.kegg.jp/kegg/kegg1.html)^14,15^, to achieve an unrivaled speed of the metabolic profiling approach. Secondly, comparing the 16S rRNA gene sequence to SILVA SSU Ref NR database (SSURef 115 NR) by BLASTN algorithm (BLAST bitscore > 1500). Thirdly, establishing a correlation matrix. Fourthly, function annotating using functional information of KEGG database annotated by UProC and PAUDA methods and the SILVA database. The OTUs of samples with SILVA database were used as reference sequences in the annotation of functional prediction. For the generation of clustered heatmaps, the pheatmap package (version 1.0.12) in R (https://cloud.r-project.org/) was used^16^.

### Ethics approval and consent to participate

This article does not contain any studies with human participants or animals performed by any of the authors.

## Results

### Sequencing data analysis

A total of 1,153,578 raw reads were obtained from 11 samples, with an average of 104,870 ± 4224 sequences for each sample (Table 1). The original reads were spliced and filtered to obtain effective data. On average, 102,823 ± 4091 raw tags, 101,953 ± 4032 clean tags, and 65,898 ± 2766 effective tags were obtained (Table 1). The average length of effective tags was 253 nucleotides (nt). The Q20 and Q30 of effect tags were 99.04 ± 0.06% and 97.44 ± 0.13% respectively (Table 1). Operational taxonomic units (OTUs) clustering and species analysis were conducted based on the effect tags. Based on 97% nucleotide sequence identity, the total number of OTUs in the 11 samples reached 28,122 with an average of 2557 ± 361 per sample.

**Table 1:** Summary of sequencing data.

Sample name	Raw PE (#)	Raw reads	Raw tags (#)	Clean tags (#)	Effective tags (#)	AvgLen (nt)	Q20	Q30	OTU
N1	103,427	103,427	101,540	100,716	63,575	253	99.14	97.66	2564
N2	109,111	109,111	106,926	106,037	67,386	253	99.06	97.5	2293
N3	110,156	110,156	108,182	107,308	69,210	253	99.04	97.45	2565
N4	106,948	106,948	104,616	103,671	69,076	253	99.01	97.42	2455
N5	98,612	98,612	96,775	96,053	62,793	253	99.06	97.5	2283
N6	96,928	96,928	95,168	94,395	61,201	253	98.97	97.21	2319
N7	105,998	105,998	103,870	102,934	63,458	253	98.95	97.25	2490
N8	105,701	105,701	103,341	102,336	67,656	253	98.99	97.38	3539
N9	105,878	105,878	103,806	102,910	67,805	253	99.03	97.44	2723
N10	108,598	108,598	106,474	105,567	67,589	253	99.03	97.44	2630
N11	102,221	102,221	100,352	99,555	65,124	253	99.11	97.58	2261

Q20 and Q30 refered to the percentage of bases with the quality score greater than 20 (sequencing error rate less than 1%) and 30 (sequencing error rate less than 0.1%) in the effective tag respectively.

### Alpha diversity analysis

**Figure 1:**
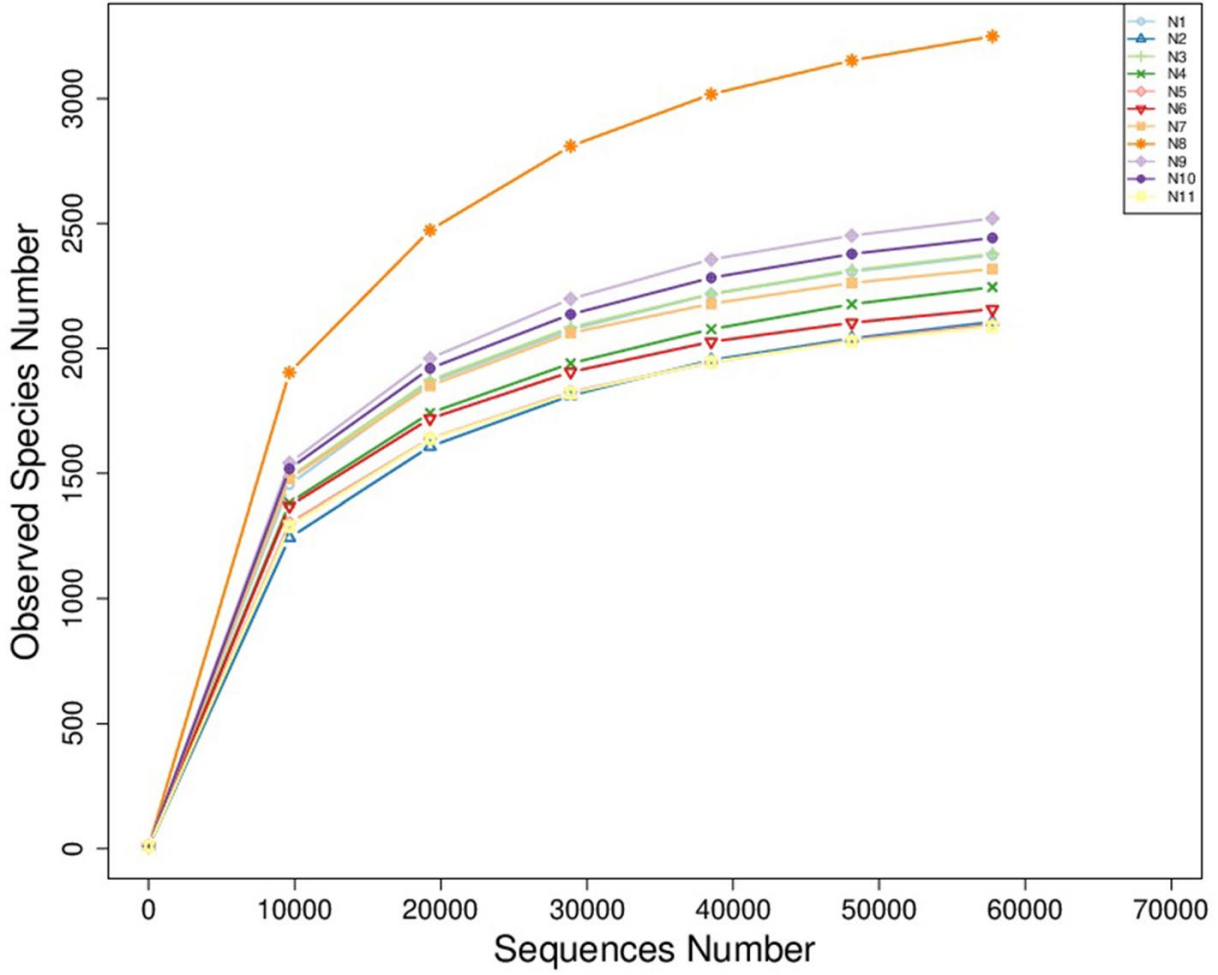
Rarefaction curves of 11 samples. *Note*: Rarefaction curves showing the number of species versus the number of sequences in per sample.

As the number of sequences increased, the rarefaction curve flattened out (Fig. 1). It was indicated that the depth of sequencing was sufficient to accurately represent the bacterial composition of the rumen (with the Good’s coverage > 99%). Furthermore, rumen microbial diversity and richness were estimated using alpha diversity index (Table 2), including Shannon, Observed species, PD whole tree, Simpson, Chao1, and ACE.

**Table 2:** Alpha diversity index of 11 samples**.**

Sample Name	Observed species	Shannon	Simpson	Chao1	ACE	Goods coverage	PD whole tree
N1	2383	8.618	0.985	2592.892	2582.93	0.994	131.876
N2	2083	8.054	0.981	2249.177	2266.789	0.994	117.533
N3	2374	8.867	0.991	2535.5	2549.487	0.994	126.113
N4	2221	8.695	0.992	2406.99	2415.315	0.994	133.613
N5	2082	8.612	0.99	2239.691	2252	0.995	111.233
N6	2159	8.778	0.991	2298.5	2309.96	0.995	113.868
N7	2310	8.952	0.992	2458.521	2464.966	0.995	123.183
N8	3270	9.435	0.994	3521.506	3552.128	0.991	182.555
N9	2517	9.056	0.994	2688.152	2707.847	0.994	138.109
N10	2433	8.991	0.993	2664.155	2626.964	0.994	130.542
N11	2103	8.261	0.978	2276.032	2289.4	0.995	113.694

Observed species: number of visually observed species (number of OTUs). Shannon: The total number of taxa in the sample and their proportion. The higher the community diversity and the more evenly distributed the species, the greater the Shannon index. Simpson: Characterise the diversity and evenness of species distributions within communities. Chao1: Estimate the total number of species contained in the community sample. ACE: Estimate the number of OTUs in the community. Goods coverage: sequencing depth index. PD whole tree: affinities of species within the community.

### Beta diversity analysis

**Figure 2:**
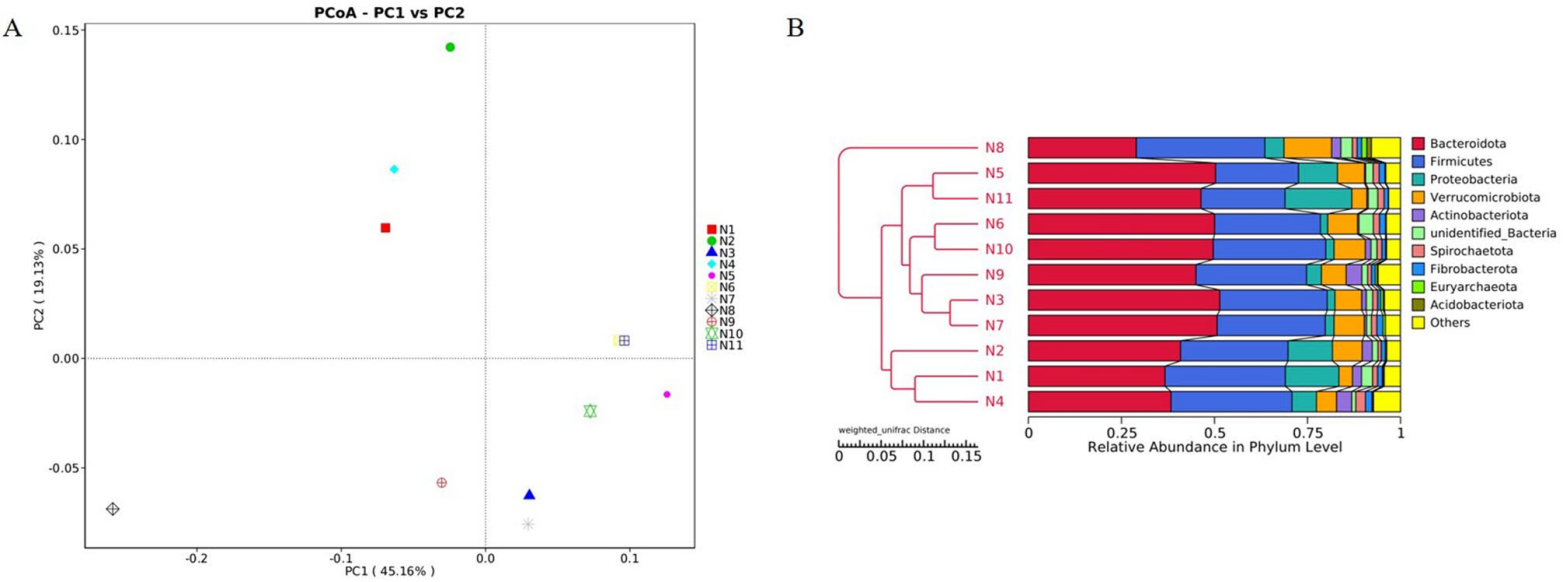
Beta diversity analysis of ruminal microbiome in 11 samples. *Note*: (**A**) Principal co-ordinates analysis (PCoA) based on weighted UniFrac distance of samples. (**B**) UPGMA clustering tree based on weighted UniFrac distance.

Beta diversity analysis was performed to carry out a comparative analysis of the microbial community composition of 11 samples. The samples with high similarity in community structure were clustered together in the plot, and the samples with large community differences would be far apart in the polt. The first and second principal coordinates of the PCoA diagram are 45.16% and 19.13% respectively (Fig. 2A). Microbial community characteristics varied widely across N8 and other samples. Similar results to PCoA mentioned above, samples were divided into three groups: (A) N1, N2, and N4; (B) N3, N5, N6, N7, N9, N10, and N11; (C) N8 (Fig. 2B).

### The bacteria composition and community of rumen in Mongolian cattle

**Figure 3:**
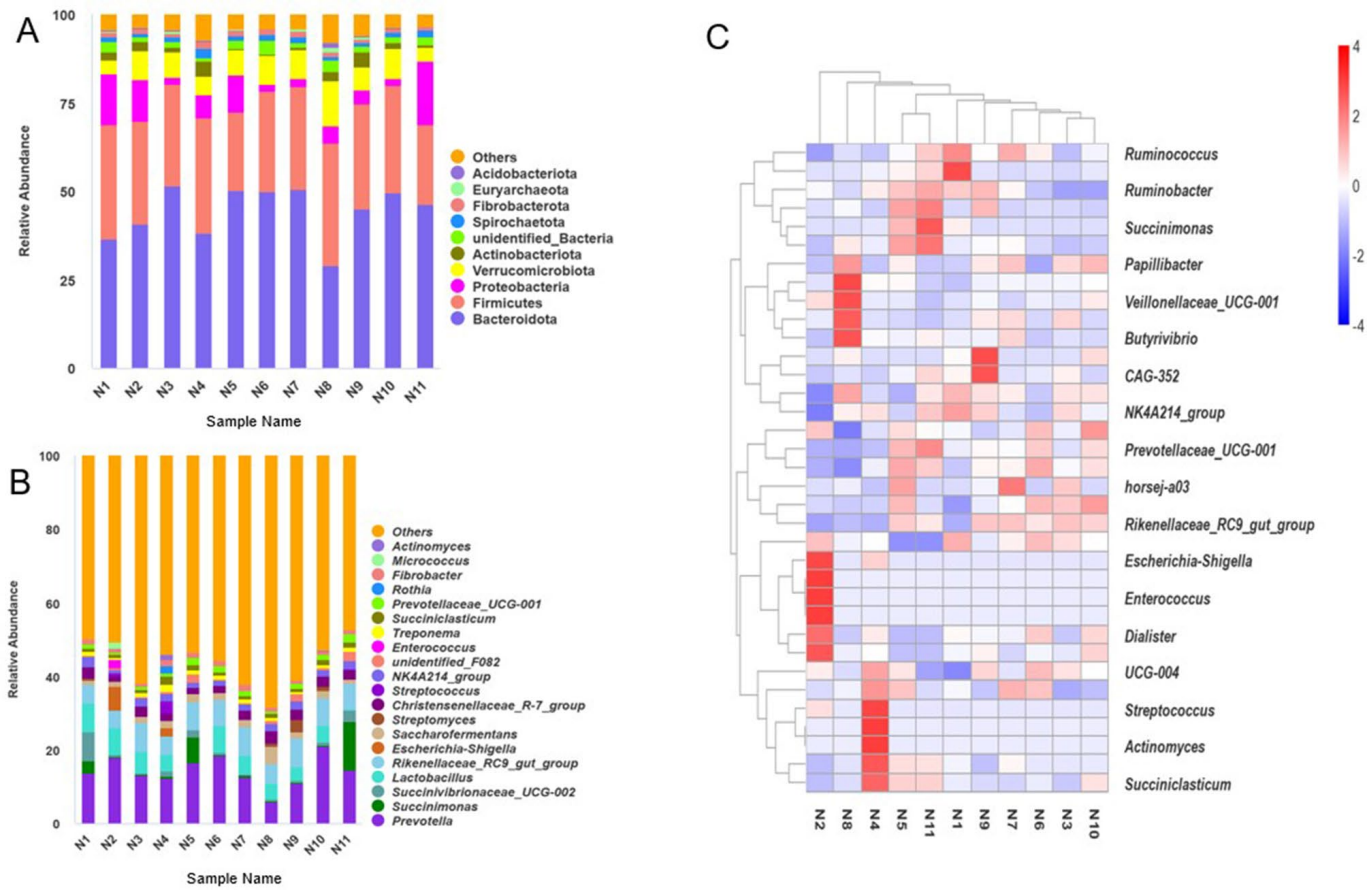
Distribution of species among the 11 samples. *Note*: (**A**) Relative abundance of rumen microbial communities at phylum level. (**B**) Relative abundance of rumen microbial communities at genus level. (**C**) Species abundance clustering heat map.

**Figure 4:**
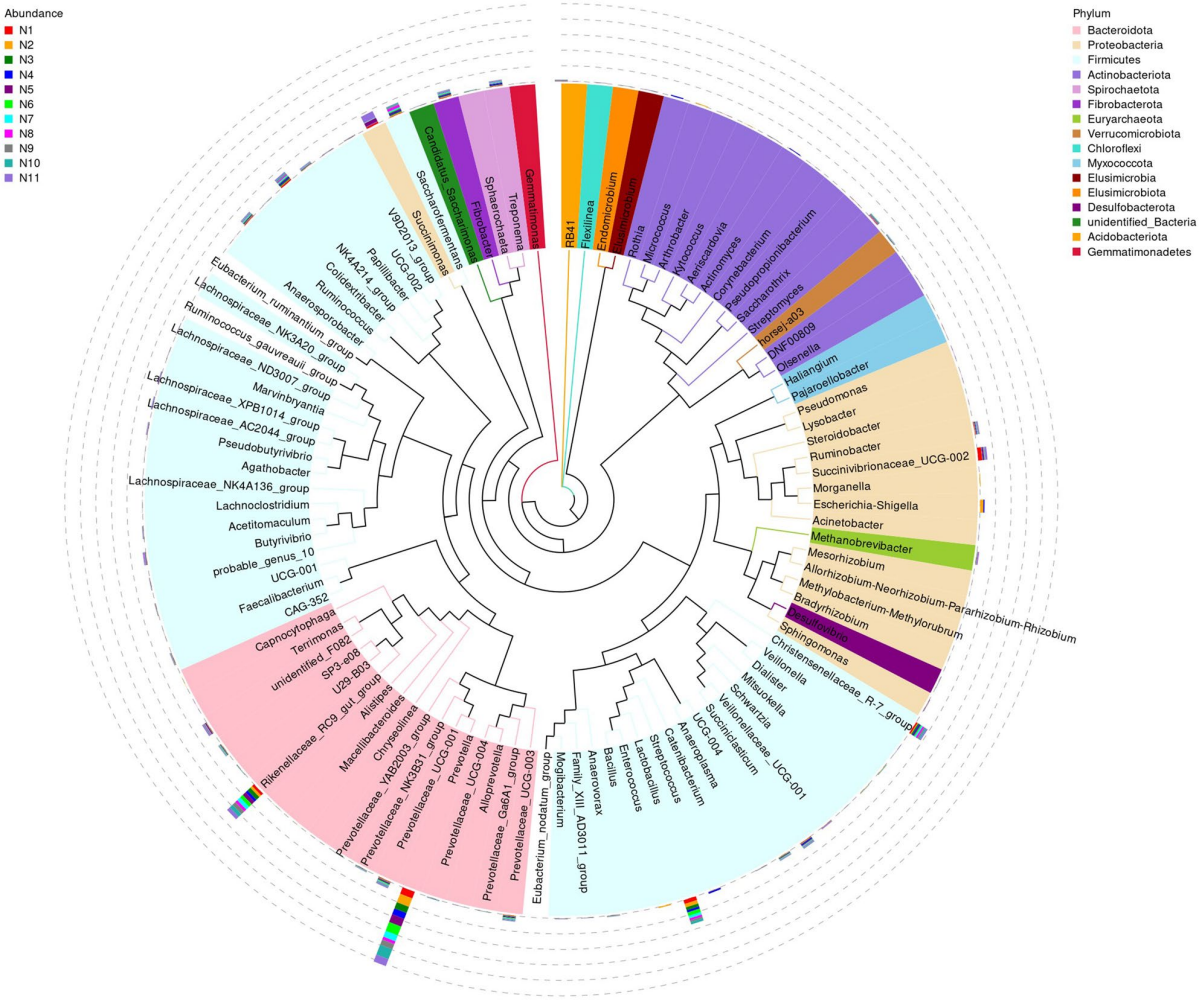
Phylogenetic tree constructed from representative sequences of genus level species. *Note*: The tree shows phylogenetic relationship of top 100 genus, the color of branches represented their corresponding phyla, and each color represented a phylum.

Cumulative bar charts of relative species abundance at the taxonomic level of the phylum (Fig. 3A) and genus (Fig. 3B) were produced to view the species with high relative abundance at different taxonomic levels and their proportion in each sample. The top ten genera in terms of relative abundance in 11 samples were *Prevotella* (14.30 ± 4.13%), *Succinimonas* (2.42 ± 4.19%), *Rikenellaceae_RC9_gut_group* (6.66 ± 1.25%), *Succinivibrionaceae_UCG-002* (1.65 ± 2.23%), *Lactobacillus* (4.57 ± 2.62%), *Escherichia-Shigella* (0.79 ± 1.96%), *Saccharofermentans* (1.98 ± 1.03%), *Christensenellaceae_R-7_group* (2.44 ± 0.60%), *unidentified_F082* (0.96 ± 0.82%), and *Ruminococcaceae_NK4A214_group* (1.88 ± 0.48%) (Figs. 3B and 4). These genera accounted for 37.66 ± 6.16% of total bacterial sequences and belonged to the three phyla with the highest relative abundance (Fig. 3A): *Bacteroidetes* (44.41 ± 7.31%), *Firmicutes* (29.07 ± 3.78%), and *Proteobacteria* (7.18 ± 5.63%). *Firmicutes* and *Bacteroides* were the core phylum in the rumen accounting for 73.48 ± 5.60%. In addition, the ratio of *Firmicutes* to *Bacteroidetes* is about 1.57.

The heat maps (Fig. 3C) was plotted to visualize the top 35 genera in terms of abundance among 11 samples. The relative abundances of *Micrococcus*, *Enterococcus*, and *Morganella Morganii* in sample N2 were higher than those in other samples. But the relative abundances of *Christensenellaceae R-7 group* and *Ruminococcaceae_ NK4A214_ group* in N2 were lower than others. The relative abundance of *Methanobrevibacter*, *Veilonellaceae-ucg-001*, and *Saccharofermentans* in sample N8 were higher than these in other samples. But the relative abundance of *Prevotrlla*, *Prevotrllaceae_Ucg-001*, and *Prevotellaceae UCG-003* were lower than these in other samples.

### Functional annotation

**Figure 5:**
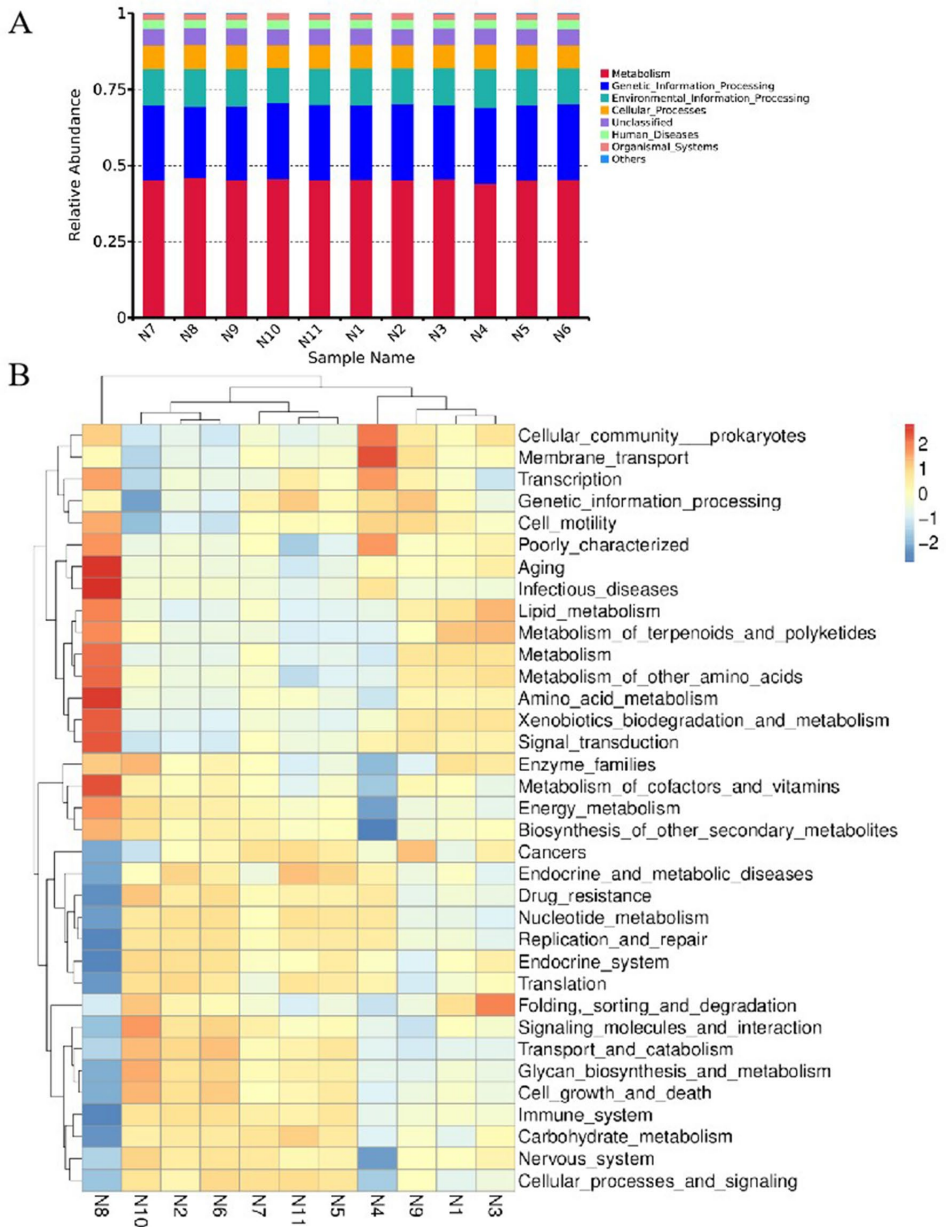
Functional forecasting through the Tax4Fun. *Note*: (**A**) Predicted function annotation relative abundance histogram at KEGG level 1. (**B**) Heat map of functional relative abundance clustering at KEGG level 2.

The Kyoto Encyclopedia of Genes and Genomes (KEGG) pathway at level 1 and 2 were performed estimated by Tax4Fun to analysis the potential function of bacteria in the 11 samples. Functional relative abundance annotation results showed that the pathways were extraordinary analogous among samples. At KEGG level 1 (Fig. 5A), the biological pathways of the samples mainly included metabolism (45.09 ± 0.39%), genetic information processing (25.13 ± 0.61%), environmental information processing (11.72 ± 0.74%), cellular processes (7.82 ± 0.15%), Unclassified (5.31 ± 0.12%), human diseases (2.86 ± 0.06%), and organism systems (2.06 ± 0.07%). The top three functional paths in terms of relative abundance accounting for more than 80%.

Hierarchical clustering heat map showing abundance information for the top 35 relative abundances of KEGG pathways in 11 samples (Fig. 5B). As the results of level 1, the functional pathways were similar among 11 samples. The KEEG pathways with high relative abundance in the 11 samples mainly included carbohydrate metabolism, replication and repair, translation, membrane transport and amino acid metabolism. The cellular community prokaryotes, membrane transport, transcription and infectious diseases were the mainly pathways in the samples N1, N4, N8, and N9. In the sample N4, the cellular community prokaryotes and membrane transport were play an important role. The folding, sorting and degradation, lipid metabolism, and metabolism of terpenoids and polyketides were the mainly metabolic pathways in the sample N3.

## Discussion

### Microbial diversity of samples

The sequencing platform and the sample numbers usually affected the quality of the 16S rRNA data. Meanwhile, if more than 10,000 reads obtained for one sample was sufficient for accurate and reliable results^17–19^. In this study the 16S rRNA gene of 11 samples were sequenced by Illumina NovaSeq platform. And every sample had obtained more than 90,000 reads. Consequently the results of the 16S rRNA data was accurate and reliable.

Alpha diversity indices included four main indices including Chao1, ACE, Shannon and PD whole tree. The Chao1 and ACE indices reflected the richness of the microbial communities. The Shannon indices reflected the diversity, and the PD whole tree reflected the genetic relationship of species in the community. The larger index values of Chao1, ACE, and Shannon indicated that the more abundant and diverse species were observed in one sample^20^. The higher alpha diversity indicated a more diverse and complex microbial composition in one rumen. It could enhance the resistance to environment and the adaptability of the host^21^. The rumen microbiota could regulate physiological processes that provided protection to the host, such as the production of antimicrobial substances and inhibition of the growth of digestive pathogens^22,23^. A good rumen microbial ecosystem can improve the growth performance of the host^24^. Dietary variation might be a major source of new microbial diversity, with alpha diversity often changing dynamically with diet^21^. In the present study, the 11 Mongolian cattle rumen samples showed similarity in terms of overall microbial diversity. The Alpha diversity indices of sample N8 was higher than those of other samples. The result indicated that sample N8 had a higher richness of species diversity.

### Microbial community structure of the Mongolian cattle

In our study, the *Bacteroidetes*, *Firmicutes*, and *Proteobacteria* were found in all 11 samples. *Firmicutes* and *Bacteroides* were the dominant flora in the rumen of mammals, they help animals digest plant-derived feed^25–27^. When the diet of adult cows was changed to a high fibre diet, the abundance of the phylum *Bacteroides* increased considerably and the composition of the rumen microbiota changes^28^. Consistently, our findings also show *Firmicutes* and *Bacteroides* account for 70% of rumen bacteria of Mongolian cattle, and the ratio of *Firmicutes* to *Bacteroidetes* was about 1.57. In the study of Elie Jam^28^, they found the abundance of *Bacteroidetes* were inversely proportional to the fat. The results found that when the fat in the blood and tissue was increased, the abundance of *Bacteroidetes* was decreased. The change of *Bacteroidetes* ratio affected the metabolic potential of the mouse gut microbiota^1^. Similarly, Turnbaugh^29^ found that the ability of the obesity microbiome to obtain energy from the diet was increased. More importantly, that this feature was transmissible: the mice had an increase in whole body fat following the introduction of microbiota positively associated with obesity in germ-free mice. The results suggested that these floras were a cause of obesity. This was of great importance for the breeding of Mongolian cattle, as the ratio of the two floras could be adjusted to regulate the fat content and thus the health and cold tolerance of the animal^30^. These results suggested that the ratio of the relative abundance of *Firmicutes* and *Bacteroides* was probably related to obesity. *Proteobacteria* was the phylum with the third highest relative abundance in the Mongolian cattle. *Proteobacteria* comprised a large number of bacteria that can catabolize feedstuff components^31^, such as fiber plant^32^. The results also demonstrated that the rumen microorganisms contained *Fibrobacteres*, accounting for nearly 1%. It was an important phylum of cellulose-degrading bacteria^33^. This phylum included one cellulase producing bacteria genus named *Fibrobacter*. And the genus named *Fibrobacter* consisted of two cellulose-degrading species named *Fibrobacter* succinogenes and *Fibrobacter* enterica.

In our study, the top ten species with relative abundance at the genus level included *Prevotella*, *Rikenellaceae_RC9_gut_group*, *Succinivibrionaceae_UCG_002*, *Lactobacillus*, *Escherichia Shigella*, *Saccharofermentans*, *Christensenellaceae_R-7_group*、*unidentified_F082*、*NK4A214_group*. These genera belong to three phyla——*Bacteroidetes*, *Firmicutes*, and *Proteobacteria*.

*Prevotella* was the genus with the highest relative abundance among rumen bacteria^34^. Although *Prevotella* couled not degrade cellulose, it could degrade other polysaccharides such as xylan. As the main proteolytic bacteria in the rumen, *Prevotella* could use peptides and ammonia as nitrogen source. Additionally, it could also produce a lot of complex enzymes to degrades starch^35^. Meanwhile, *Prevotella* were associated with the production of propionate in the rumen^24^. Propionate was negatively correlated with methane production^26,36^. This showed that an increase in the relative abundance of *Prevotella* favored a reduction in methane emissions. Furthermore, it had an important role to play in reducing greenhouse gas emission from ruminants.

In the present study, the relative abundance of *Succinimonas* and *Succinivibrionaceae_UCG_002* was accounting for 4%. *Succinimonas* were anaerobic organisms that break down starch, ferment glucose, maltose and dextrin. The metabolites were a large amount of succinic acid and a small amount of acetic acid^37^. The relative abundance of *Succinivibrionaceae_UCG_002* was higher than others in the *Succinivibrionaceae* family. The abundances of all the *Succinivibrionaceae* members were positively correlated with the emission of methane. Because its members mainly produced succinate, that competed with methane production, thus they reduced the release of hydrogen^38,39^. Some studies have found that the presence of *Succinivibrionaceae_UCG_002* and *Succinivibrio* was inversely proportional to feed efficiency^40,41^. Some species of the *Succinivibrionaceae* family from the tammar wallaby (*Macropus eugenii*) manipulated methane and therefore it drew attention to methane emissions in ruminant livestock^42^.

The genus with the second highest relative abundance in 11 samples was *Rikenellaceae_RC9_gut_group*. And the lower pH would reduce the amount of this genus. Meanwhile the relative abundance varies by sex, with lower abundance of the genus in female cows^43^. A recent study^36^ found the abundance was also associated with rumen epithelial morphology. The rumen epithelium was important for the absorption of volatile fatty acids from feed digestion, with approximately 50–80% of volatile fatty acids (VFAs). The VFAs were absorbed directly by the epithelium^44^. Overall,* Rikenellaceae_RC9_gut_group* in the rumen has an important role in the degradation of crude fibre and in the morphological structure of the rumen epithelium^35^.

*Lactobacillus* was composed of over 170 species and 17 subspecies^45^. As a typical bacterium of *Lactobacillus, Lactobacilli* had long been used to make dairy products such as cheese and yogurt. They also had a high tolerance for very low pH conditions, especially those used to ferment foodstuffs such as mustard, cabbage, and olives. In the gut, *Lactobacillus* adhesion to the mucus layer of the gut wall was mediated by a protein surface layer called the S-layer. In addition, some strains of *lactobacilli* produced antioxidants. Ljungh and Wadström^46^ also reviewed that probiotic potential of lactobacilli, was the ability for immunomodulate human cells to achieve an anti-inflammatory response. In addition, *Lactobacilli* could produce strain-specific bacteriocins and bacteriocin-like products that could inhibit the growth of other organisms^47^. *Lactobacillus* was a partly anaerobic or strictly anaerobic bacterium, and it was involved in the hydrolysis of proteins and lipids. In particular, it could continue to multiply after participating in the formation of cheese and thus produced contamination^48^. As *Lactobacillus* fermenting glucose could release lactic or acetic acid, which produced an acidic environment that inhibited the growth of some harmful bacteria. It was helpful for the health of the host. In addition to that, *Lactobacillus* could also alleviate respiratory diseases and regulate respiratory immunity^49^ and provide some relief from the symptoms of cardiovascular disease^50^. But the genus *Lactobacillus* was not only a beneficial organism, but can also act as a pathogen for serious infections. If *Lactobacillus* entered the bloodstream, it might be associated with sepsis in immunocompromised patients^46^.

*Escherichia Shigella* is a facultative anaerobic microorganism as well as a pathogenic bacterium. The rapid increase of *Escherichia Shigella* abundance in the intestine and stomach is one of the signs of IgAN (IgA nephropathy) production, which leads to the imbalance of intestinal ecology of patients and the decline of immunity^51^. The study of Lee^52^ showed that diarrhoea caused by *Escherichia-Shigella* is not associated with changes in children's weight, but can slow height growth in children. The presence of large amounts of *Escherichia-Shigella* is detrimental to the health of the host, there is a small relative abundance of *Escherichia-Shigella* among samples, accounting for less than 0.00002%. It shows that the 11 samples in this study are all from healthy Mongolian cattle.

Many studies had shown that the genus *Christensenellaceae R7 group* was found in the intestinal tract and played an important role in amino acid and lipid metabolisms^53^. It was recently found *Christensenellaceae was* associated with the health of digestive system for human and mice^54^. The *Christensenellaceae_R-7_group* was an important member of the *Christensenellaceae*, and *Christensenellaceae* was importantly associated with mammal health. The relative abundance of *Christensenellaceae* was inversely proportional to fat content, with higher abundance being beneficial to health. Thus the relative abundance of *Christensenellaceae* was higher in centenarians^53^. The fermented complete feed significantly affected the microbial diversity in pig manure, with the most affected genera including *Christensenellaceae_R-7_group*, whose relative abundance was significantly reduced compared to the control group^55^. Yang’s studies indicated that the abundance of the *Christensenellaceae_R-7_group* was increased in the rumen as the content of n3-poly unsaturated fatty acids in longissimus dorsi muscle was increased^56^. And it might play an important role in the low-density fatty acids metabolism for mammals health. In present study, the relative abundances of *Christensenellaceae_R-7_group* were very similar among 11 samples, averagely accounting for 4.5%.

### Functional pathways of rumen bacteria in the Mongolian cattle

According to the function prediction analysis of KEGG, it had found that the rumen bacteria in Mongolian cattle play roles in the metabolism of gene, cell, carbohydrate, amino acid, energy and vitamin. All findings indicated that bacteria played an important role in nutrient digestion and absorption of ruminants. The fiber was digested through the bacteria in the rumen into volatile fatty acids, such as acetic acid, propionic acid, and butyric acid^57,58^. Meanwhile, the bacteria could convert the nitrogen-containing substances into amino acids and provided the nitrogen source for ruminants. Consequently, the abundance and composition of the rumen bacterial population was closely correlated with the health of the ruminants^59,60^. Research had shown that the diversity and abundance of rumen bacteria was closely related to the composition and type of feed^60^. Highly nutritious feeds could reduce the variety of rumen microorganisms and even caused subacute ruminal acidosis (SARA), which was detrimental to the health of cattle and could even cause death^61^. Therefore, ruminal microbial composition could be controlled through scientific feed ratios to improve the stability of the rumen microbial community and increase ruminant production^62^. For example, when the ratio of concentrate to roughage in feeds was changed, it could maximize productivity, reduce energy losses and decrease the methane emission^63^.

## Conclusion

Our findings showed that there was higher diversity, richness, and evenness of microbial communities in the rumen. The predominant phylum were *Bacteroidetes* and *Firmicutes* in the rumen, *Prevotella* was the most predominant genus in the rumen. And the biological pathways in microbial communities of rumen mainly included metabolism, environmental information processing, and genetic information processing. Nevertheless, the functional profiles of 11 samples were merely a prediction; detailed analyses are still needed to elucidate this aspect. Further studies are warranted to determine the contributions of the bacterial taxa and metabolic pathways to the health, development, and physiology of cattle.

Inner Mongolia is the region with the larg number of cattle in China. Meanwhile, understanding the rumen microbial composition of Mongolian cattle is an important guide to the health of cattle. In our study, we innovatively collected rumen samples from Mongolian cattle in Xilingol League, which was rarely seen in previous studies. The experimental results showed that the rumen microbial diversity in the samples was high, including some rare and uncultured microbes. These microbes play important ecological functions in the rumen of Mongolian cattle, and were of great significance for understanding the integrity and stability of rumen microbial communities. Moreover, exploring the rumen microbial diversity was important for enriching the microbial resource. It could provide guidance for feeding cattle. For example, feed formulation could be adjusted according to the species and number of rumen microorganisms to improve feed utilization. And the feeding environment could be improved according to the ecological characteristics of rumen microorganisms to enhance the health status of Mongolian cattle. Functional gene could also be characterized, which laid the foundation for elucidating the metabolic pathways for degrading cellulose and fat, and so on. Consequently, cellulose-degrading bacterial would been collected and cellulose genes would been characterized in our laboratory from Mongolian cattle according to the results of this study.

## Data Availability

The raw sequencing data have been deposited on the Sequencing Reads Archive (SRA), and SRA accession number is PRJNA955685 (https://www.ncbi.nlm.nih.gov/sra/PRJNA955685).
